# Interpol review of forensic image and video analysis, 2022-2025

**DOI:** 10.1016/j.fsisyn.2026.100697

**Published:** 2026-06-16

**Authors:** Zeno Geradts, Stijn van Lierop, Meike Kombrink

**Affiliations:** a Netherlands Forensic Institute, Laan van Ypenburg 6, Den Haag, 2497 GB, Netherlands; b University of Amsterdam, Institute for Informatics, Science Park 900, Amsterdam, 1098 XH, Netherlands

## Abstract

The field of forensic image and video analysis is undergoing a transformation, driven by technological innovation and an increasing demand for scientific proof. This review provides an analysis of the state-of-the-art from 2022 to the present, the progress and challenges across key domains. The amount of digital media has made images and videos central to legal investigations. The same technologies that create this evidence also enable sophisticated manipulation, posing a significant threat to its integrity. Deep learning has emerged as a dominant paradigm, offering powerful new capabilities but also introducing challenges related to transparency and validation. This paper examines the maturation of core forensic practices, including the critical re-evaluation of Photo Response Non-Uniformity (PRNU) for source camera identification in an era of computational photography, and the evolution of image enhancement and authentication techniques. We explore the rapid advancements in content analysis, particularly in deep learning-based methods for detecting digital forgery and steganography, highlighting the ongoing "arms race" between manipulation and detection. In biometric and scene-based analysis, we describe the shift in facial comparison towards a probabilistic likelihood ratio framework for expressing evidential value and review the current state of forensic gait analysis and photogrammetry, including its application in vehicle speed estimation. Throughout this review, we emphasize the central tension between the pace of technological advancement and the crucial, ongoing efforts by international bodies such as ENFSI, SWGDE, FISWG, and Interpol to establish the robust standards, best practices, and validation frameworks necessary for ensuring the admissibility and reliability of digital evidence in court. The paper concludes by key trends and outlining future directions, focusing on the needs for explainability, robustness, and continued international collaboration to bridge the gap between innovation and forensically sound application.

## 1 Introduction

The digital age has fundamentally changed forensics. The amounts of image and video recording devices—from smartphones and body-worn cameras to vast networks of closed-circuit television (CCTV)—has resulted in a huge volume of digital media being presented as evidence in criminal and civil proceedings [1,2]. This amount of digital evidence offers potential for investigation, providing visual records of events that can be important in establishing facts and determining outcomes [3,4]. However, this technological surge has two sides. The same accessible and powerful software that enables the creation and sharing of media also facilitates sophisticated manipulation, capable of altering content in ways that are often imperceptible to the naked eye. This reality poses a direct threat to the foundational principles of evidence integrity and authenticity, creating a crisis of trust in digital media [2,[5], [6], [7]].

At the heart of the recent evolution in multimedia forensics is the rise of deep learning, a subfield of artificial intelligence [8] that mimics human learning processes to automatically extract hierarchical features and make predictions from vast datasets [9,10]. Deep learning has become a transformative force, with applications in nearly every subdomain of forensic image and video analysis, from enhancement and authentication to manipulation detection and biometric identification [6,9]. These methods have demonstrated the capacity to surpass the performance of traditional algorithms and, in some cases, human experts [11].

This rapid technological advancement has created fundamental tension in the forensic community. While academic research produces a constant stream of powerful new algorithms, the legal system demands methods that are not only accurate but also transparent, repeatable, and rigorously validated. This has led to the establishment and increasing prominence of international working groups and standards bodies dedicated to navigating this complex terrain. Organizations such as the European Network of Forensic Science Institutes (ENFSI), the Scientific Working Group on Digital Evidence (SWGDE), the Facial Identification Scientific Working Group (FISWG), and the Organization of Scientific Area Committees (OSAC) for Forensic Science are working to create the best practices, guidelines, and formal standards required to bridge the "validation gap" between cutting-edge research and its responsible application in the courtroom [[12], [13], [14], [15]]. Global law enforcement bodies like Interpol are also playing a crucial role, not only by operating large-scale biometric databases but also by promoting policy frameworks for the ethical use of these powerful technologies [16].

This review provides a survey of the field of forensic image and video analysis, focusing on developments from 2022 to the present. It begins by examining practices of image enhancement and authentication, where the promise of AI is tempered by concerns over evidentiary validity. It then moves to a critical evaluation of source identification, questioning the long-held reliability of PRNU in the face of modern computational photography. The paper subsequently delves into the "arms race" of content analysis, reviewing the latest deep learning techniques for forgery and steganography detection. Following this, it covers the analysis of scene content, including the maturation of facial comparison through the likelihood ratio framework, the state of forensic gait analysis, and the application of photogrammetry to tasks like vehicle speed estimation. Finally, the review synthesizes the critical role of international standardization efforts and outlines the key challenges and future directions for the field, emphasizing the need for methods that are not only powerful but also forensically reliable and legally admissible.

The review is based on searching on google scholar and scopus on (image? or video?) and forensic? and also several separate searches on each separate topic of the review. Since we have over 5000 papers in the last 3 years, a selection of hand picked papers are used in this review mostly based on title and abstract. Those papers are read in depth. In some cases references before 2022 are used since they are the leading references for research after this time. This review will provide a global overview of the field, and we will never be complete due to the number of papers that are published, also we limit our searches mostly to English sources. Though we expect to have to main developments in the field in this review. Papers on deep fake detection are handled in a separate paper by [17].

## 2 Enhancement and authentication

Before any substantive analysis of the content of an image or video can occur, two processes are often requested: enhancement, to improve the quality of the media for interpretation, and authentication, to establish its credibility as a piece of evidence. These steps are governed by strict forensic principles to ensure that the integrity of the original evidence is maintained and that any procedures applied are documented and defensible. Recent years have seen significant academic advancements in these areas, largely driven by deep learning, but these innovations have also introduced new and complex challenges to the forensic validity of the evidence.

### 2.1 Image and video enhancement

Forensic image and video enhancement refers to any process intended to improve the visibility or interpretability of specific details within digital media for an intended purpose, such as identifying a person, reading a license plate, or reconstructing an event sequence [12]. While traditional techniques like histogram equalization and gamma correction remain in use due to their simplicity and computational efficiency, the field has increasingly shifted towards sophisticated deep learning-based approaches in academic literature [[18], [19], [20]].

Deep learning models, particularly those based on Convolutional Neural Networks (CNNs), have demonstrated remarkable success in complex enhancement tasks like low-light enhancement and super-resolution (increasing the spatial resolution of an image). Architectures such as UNet-inspired encoder-decoders, often augmented with attention modules to focus on relevant features and suppress noise, are now commonplace [18]. Generative Adversarial Networks (GANs), such as the Enhanced Super-Resolution GAN (ESRGAN), have shown the ability to generate highly realistic details in upscaled images [21,22]. These models are typically trained on large-scale benchmark datasets, such as LOL (Low-Light) or SID (See-in-the-Dark), to learn the complex mapping from degraded to high-quality images [18].

However, the power of these generative models raises a critical question of forensic validity. A core principle of forensic enhancement is that the process must not create information that was not present in the original data. This has led to an important distinction between "restoration-focused AI" and "generation-focused AI" [23]. Restoration-focused methods operate within a defined mathematical model to reverse known degradation processes like noise or blur, effectively restoring the original signal. In contrast, generation-focused methods, particularly those used for super-resolution, may enhance semantic content by "hallucinating" or generating plausible but not necessarily factual details to fill in missing information. This poses a significant risk to evidentiary integrity, as the enhanced image may contain details that are artifacts of the AI model rather than true representations of the original scene.

The potential for such algorithms to negatively impact forensic analysis is not merely theoretical. A recent study by Macarulla Rodriguez et al. (2024) found that preprocessing facial images with the CodeFormer superresolution model, a state-of-the-art tool, *undermined* the reliability of a subsequent forensic face recognition task. The process, intended to improve image quality, actually increased the error rate of the recognition system, demonstrating that enhancement can be detrimental to other forensic examinations [24]. This underscores the caution required when applying advanced AI enhancement tools in a forensic workflow.

Recognizing these risks, international standards bodies have established clear guidelines. The ENFSI *Best Practice Manual for Forensic Image and Video Enhancement* and SWGDE guidelines both emphasize that the original evidence must be preserved as an unaltered master copy (e.g., as a write-protected file or verified copy) and that all processing steps must be meticulously documented in an image processing log [12,25]. This documentation ensures that any enhancement process is transparent, repeatable, and can be scrutinized in court.

### 2.2 Image and video authentication

Image authentication is the process of substantiating that digital data is an accurate representation of what it purports to be [26]. It is a distinct concept from file integrity. Integrity can be verified with a cryptographic hash function (e.g., SHA-256), which confirms that a file is a bit-for-bit identical copy of another; however, this proves nothing about the authenticity of the content *within* the file [26,27]. An image can have perfect integrity (the file has not been altered since its creation) but be entirely inauthentic (e.g., a composite of multiple images).

The authentication process is a multi-faceted examination that scrutinizes the image's structure, content, and chain of evidence. This methodology is heavily guided by best practice documents from SWGDE and OSAC, which provide a robust framework for the examination [25,27]. The typical workflow involves:

● **Image Structure Analysis:** This involves a technical examination of the file's underlying data and metadata. Analysts inspect the file format for consistency with its purported source, examine header and footer information at the hexadecimal level, and analyze metadata such as the Exchangeable Image File Format (EXIF) data [27]. EXIF data can contain a wealth of information, including camera make and model, settings (exposure, focal length), date and time stamps, and sometimes even GPS coordinates. However, analysts must be cautious, as metadata can be easily stripped or altered [27].
● **Image Content Analysis:** This is an examination of the visual information within the image itself. The analyst looks for inconsistencies that may indicate manipulation. These include anomalies in lighting (e.g., multiple light sources creating inconsistent shadows), incorrect perspective or scale between objects, unnatural focus or depth-of-field, and tell-tale artifacts from editing processes like cloning, splicing, or compositing [27,28]. The consistency of compression artifacts (e.g., JPEG blocking) and noise across the image is also a key indicator.

There exists an inherent and growing conflict between the goals of enhancement and authentication. The powerful AI-based enhancement techniques discussed previously can inadvertently destroy or alter the very forensic traces that an authentication analysis relies upon. For instance, a super-resolution algorithm might smooth over the subtle inconsistencies in JPEG compression blocks that would otherwise signal a splicing manipulation. A denoising algorithm could remove the unique sensor noise pattern used for source identification (discussed in the next section). The negative impact of the CodeFormer model on facial comparison reliability is a prime example of this conflict [24]. An enhancement process intended to make a face clearer for a jury might fundamentally alter the underlying data, rendering a subsequent technical authentication impossible.

This necessitates a strict, documented, and logical workflow, as outlined by both SWGDE and ENFSI [12,15]. The main rule is to keep the original, unaltered evidence. Any enhancement should be considered a terminal process, applied judiciously and only after other technical analyses (like authentication) are complete. The forensic report must transparently document every tool and process used, allowing for independent review of the entire analytical chain.

## 3 Content analysis and manipulation detection

Beyond verifying the origin and basic authenticity of a digital file, forensic analysis often focuses on its semantic content. This involves two critical tasks: detecting manipulations, where parts of an image have been altered, added, or removed to create a false narrative, and steganalysis, the process of uncovering data that has been intentionally hidden within the media. In recent years, both domains have become an "arms race" between the creators of sophisticated forgeries and the developers of detection tools, with deep learning at the epicentre of this conflict.

### 3.1 Detecting digital forgery

The ease of access to powerful image editing software has made the creation of visually seamless forgeries a widespread problem ([2]; Tech Science Press, 2024). Detecting these forgeries, which include common types like splicing (pasting a region from one image into another), copy-move (copying and pasting a region within the same image), and object removal, is a primary goal of forensic analysis.

While traditional methods based on statistical inconsistencies exist, the research landscape since 2022 has been dominated by deep learning approaches [6]. This shift is a direct response to the limitations of older methods in detecting forgeries created with modern, sophisticated tools. The core of these new methods are typically a Convolutional Neural Network (CNN) and vision transformers, which are trained to learn the subtle artifacts and inconsistencies introduced by the manipulation process.

Various architectures have been proposed. Foundational models like VGG16 and ResNet are often adapted for the task, sometimes in combination with other techniques [29,30]. For instance, some methods first apply Error Level Analysis (ELA)—a technique that highlights differences in JPEG compression levels within an image—and then feed the resulting ELA map into a CNN to improve detection accuracy [1,31]. Hybrid models, such as a CNN combined with a Long Short-Term Memory (LSTM) network, have also been explored to capture both spatial artifacts and sequential patterns within the image data [29]. Table 2 provides an overview of these common architectures.

The performance of these deep learning models is critically dependent on the quality and diversity of the datasets used for training. Several public benchmark datasets are commonly used in the literature to train and evaluate forgery detection models, including CASIA (v1 and v2), Columbia Uncompressed Image Splicing Detection, and the MICC series (F220, F600, F2000), which specifically feature copy-move forgeries with additional transformations like scaling and rotation [2,31,32].

While many academic papers report very high detection accuracies, often in the range of 92% to 99% on these specific datasets [1,30,31], a significant challenge remains generalization. Models trained on one type of forgery or dataset often perform poorly when tested on different manipulation types or on "in-the-wild" images sourced from social media platforms, which undergo unknown and often severe compression and resizing [30]. This gap between laboratory performance and real-world effectiveness is a key area of ongoing research.

### 3.2 Uncovering hidden data: steganalysis

Steganography is the art and science of hiding information within an innocuous-looking cover file, such as an image, in a way that conceals the very existence of the communication [33]. This technique is used for malicious purposes ranging from industrial espionage and information theft to coordinating terrorist activities and distributing child sexual abuse material, making its detection—a process known as steganalysis—a critical task for law enforcement and intelligence agencies [33,34].

Steganalysis techniques can be broadly categorized. Traditional methods include signature-based analysis, which looks for known file headers or footers left by specific steganography tools, and statistical analysis, which detects subtle changes in the statistical properties of an image (e.g., its color histogram) caused by data embedding [35]. While effective against older or simpler steganography methods, these approaches are often limited to known algorithms and can be defeated by more advanced, adaptive techniques that minimize statistical disturbances [36].

Consequently, modern steganalysis has, like forgery detection, moved partly towards deep learning. CNN-based steganalyzers (e.g., SRNet, YeNet) are designed to automatically learn the minute, high-dimensional statistical traces left by embedding algorithms, even at very low payloads ([34,37,38]; Zhen et al., 2024). These models have proven to be more robust and adaptable to new or unknown steganography methods than their handcrafted predecessors [37,38].

The nature of this "arms race" has prompted significant international collaboration. A leading example is the European Union's Horizon 2020 project, UNCOVER (Development of an efficient steganalysis framework for uncovering hidden data in digital media). This project brought together a consortium of 22 partners, including law enforcement agencies (like the Netherlands Forensic Institute), universities, and technology companies, with the explicit goal of developing an efficient and comprehensive steganalysis framework [33,39]. UNCOVER aimed to consolidate knowledge of existing tools, improve detection methods across all media types (image, video, audio, and text), and deliver a user-friendly toolkit directly to forensic practitioners, demonstrating the strategic importance placed on combating this threat [40].

Despite these advances, significant challenges remain. One of the most difficult problems in deep learning-based steganalysis is the "cover-source mismatch," where a model trained on images from one source (e.g., a specific set of cameras or a particular dataset) performs poorly when tested on images from a different source due to variations in their intrinsic statistical properties [41]. Furthermore, the robustness of steganalysis and steganography models to common image transformations like compression, resizing, and noise addition—which are ubiquitous in real-world scenarios, especially with images shared online—is a critical area of ongoing research [37,38]. The arms race dynamic is ever-present. As detectors become more sensitive, steganographers develop new embedding algorithms specifically designed to evade them, requiring a continuous cycle of research, development, and adaptation from the forensic community.

## 4 Source identification: the evolution of device fingerprinting

A fundamental question in many investigations is determining the origin of a digital image or video. Source camera identification, the process of linking images and video to the specific device that captured it, has long been a cornerstone of multimedia forensics. For years, this field was dominated by a single, powerful technique: Photo Response Non-Uniformity (PRNU). However, recent research, driven by the complexities of modern imaging devices, has begun to critically re-evaluate the reliability of PRNU and explore new, more robust methods for device fingerprinting.

### 4.1 Photo Response Non-Uniformity (PRNU): the established fingerprint

PRNU is a subtle, noise-like pattern that is unique to each digital camera sensor. It arises from microscopic imperfections in the silicon wafer during the manufacturing process, which cause individual pixels to respond slightly differently to light [42,43]. This pattern is deterministic, stable over time, and acts as a unique, intrinsic fingerprint for that specific sensor [42]. The PRNU signal is weak, multiplicative (its intensity scales with the intensity of the light hitting the sensor), and resides primarily in the high-frequency components of an image [43,44]).

The standard forensic workflow for PRNU-based source identification involves two main stages [45,46]. First, a reference camera fingerprint, or "reference PRNU," is estimated by averaging the noise residuals from a set of images known to have been taken by the camera in question. Second, the noise residual is extracted from a questioned image. Finally, a correlation metric, most commonly the Peak-to-Correlation Energy (PCE), is calculated between the questioned image's noise and the reference PRNU. A high PCE value indicates a strong correlation, providing strong evidence that the questioned image was captured by that specific camera.

### 4.2 A critical evaluation of PRNU's reliability

For over a decade, PRNU was widely considered a "gold standard" for camera source identification. However, a growing body of research since 2022 has highlighted significant limitations and vulnerabilities, challenging its universal reliability, especially with media from modern devices. This critical re-evaluation is essential for the responsible application of the technique in court.

One of the most significant challenges comes from the rise of computational photography, particularly in smartphones. Modern devices employ heavy in-camera image processing pipelines to improve visual quality. These processes, which can include advanced noise reduction, sharpening, and multi-frame fusion (e.g., for HDR imaging), can introduce non-unique artifacts (NUAs) into the final image [47]. These NUAs can interfere with or even overwrite the subtle PRNU signal, reducing its distinctiveness. This has been shown to lead to a high rate of false alarms (false positives), especially when comparing images from different devices of the same model and brand [47,48]. Also one should take care of multiple cameras on one smartphone.

Video evidence presents its own challenges. Heavy compression, which is standard for video, can significantly attenuate the high-frequency PRNU signal [44]. Furthermore, electronic image stabilization (EIS), a common feature in modern smartphones and cameras, works by digitally cropping and warping frames to counteract motion. This process breaks the fixed spatial relationship between the scene content and the sensor pixels, effectively misaligning the PRNU pattern and making standard correlation-based detection unreliable [44,49].

The fragility of the PRNU signal is another key concern. Because it resides in the high-frequency band, it is highly susceptible to any image processing that affects these frequencies. This includes not only incidental processing like aggressive JPEG (re)compression but also deliberate counter-forensic attacks. Simple low-pass filtering or geometric transformations like resizing can effectively remove or desynchronize the PRNU, making source identification impossible (Gupta et al., 2022).

Finally, even under ideal processing conditions, PRNU reliability can be compromised by non-ideal image acquisition. A recent study by Flenner and Farid (2024) demonstrated that off-nominal exposures have a dramatic effect on error rates. They found that under-exposed (dark) images led to a false-positive rate of approximately one in two hundred—a rate often considered unacceptable in a forensic context. Conversely, over-exposed (bright) images resulted in a significant drop in the true-positive rate, meaning many true matches were missed (Flenner & Farid, 2024). Compounding these issues, the PRNU extraction process itself can be imperfect, with low-frequency interference from scene content and other sensor artifacts contaminating the estimated fingerprint (Wang et al., 2024). Table 1 summarizes these critical limitations.

**Table 1:** Comparison of deep learning architectures for image manipulation detection.

Architecture/Method	Target Forgery Type	Key Principle	Common Datasets	Strengths & Limitations	Key References
**CNN (e.g., VGG, ResNet)**	Splicing, Copy-Move	Learns hierarchical spatial features and inconsistencies (e.g., noise, compression artifacts) directly from image pixels.	CASIA, Columbia, MICC	High accuracy on specific forgery types; can be prone to overfitting and poor generalization to unseen manipulations.	Reddy & Rao (2023) [32],
**ELA + CNN**	General/Universal	Uses Error Level Analysis (ELA) to pre-process the image, highlighting differences in JPEG compression levels, which are then classified by a CNN.	CASIA, Custom Datasets	Effective at detecting manipulations involving recompression; performance depends heavily on the quality of the ELA map.	Hussain et al. (2023), Al-Sarem et al. [31]
**CNN-LSTM**	Copy-Move, Splicing	Combines a CNN for spatial feature extraction with a Long Short-Term Memory (LSTM) network to analyze sequential relationships between image patches.	MICC, Custom Datasets	Can capture both spatial and contextual information; may be more complex to train and computationally expensive.	Shelar, Sharma, & Rawat [29]
**Vision Transformer (ViT)**	General/Universal	Divides the image into patches and processes them as a sequence, allowing it to capture long-range dependencies and global context.	FaceForensics++, Various	Potentially better at capturing global inconsistencies; requires very large datasets for effective training.	Coccomini et al. [50]

**Table 2:** Critical evaluation of PRNU as a source identifier.

Limitation/Vulnerability	Description of Impact	Affected Media	Key References
**In-Camera Processing**	Heavy processing in modern devices introduces non-unique artifacts (NUAs) that reduce PRNU distinctiveness, leading to high false positive rates between same-model devices.	Modern Smartphones (e.g., Samsung, Huawei), High-end Cameras	Bhat et al. [47], Baracchi et al. [48],
**Video Compression & Stabilization**	Lossy compression attenuates the high-frequency PRNU signal. Electronic Image Stabilization (EIS) spatially misaligned the pattern, defeating correlation-based matching.	Highly Compressed Video, Videos from devices with EIS	Pasquini et al. (2022), Taspinar et al. [49]
**Post-Processing & Counter-Forensics**	As a high-frequency signal, PRNU is vulnerable to removal by low-pass filtering, geometric transformations (resizing, cropping), and deliberate anti-forensic attacks.	All processed images and videos	Gupta et al. (2022)
**Non-Ideal Exposure**	Under-exposed images significantly increase the false positive rate. Over-exposed images significantly decrease the true positive rate.	Images/videos with poor lighting conditions	Flenner & Farid (2024)
**Low-Frequency Interference**	The PRNU extraction process can be contaminated by low-frequency signals from scene content and other sensor artifacts, reducing fingerprint quality.	All images and videos	Wang et al. (2024)

### 4.3 Beyond PRNU: the search for robust fingerprints

The recognized limitations of PRNU have caused a new wave of research aimed at discovering more robust device fingerprints. The most promising direction involves a paradigm shift away from handcrafted features towards data-driven methods that use deep learning to learn device-specific fingerprints directly from image data (Gupta et al., 2022; [44]).

These emerging techniques, often employing CNNs, have uncovered novel fingerprints that are distinctly different from PRNU. A key finding is that these learned fingerprints often reside in the low- and mid-frequency bands of an image (Gupta et al., 2022; [44]). This is a significant advantage, as these frequency bands are much more resilient to the aggressive JPEG compression and processing that typically degrades the high-frequency PRNU signal. Furthermore, these new fingerprints are described as being stochastic, globally present, and location-independent, which resolves the critical spatial synchronization problem that hinders PRNU analysis in videos with digital stabilization or in geometrically transformed images (Gupta et al., 2022).

The field of source identification is clearly at a turning point. While PRNU remains a valuable tool, its status as a universal "gold standard" is no longer tenable, particularly for evidence originating from the very devices—smartphones—that are most common in casework. The future of the discipline will likely involve a hybrid approach, where analysis of PRNU is supplemented or even replaced by these new, more robust learned fingerprints. This evolution, however, brings its own challenges. The traditional PRNU method, based on a simple correlation, is relatively transparent. The new deep learning-based methods are inherently less transparent which re-emphasizes the critical need for rigorous validation, standardized reporting, and, if possible, the development of explainable AI (XAI) techniques before they can be widely adopted in forensic practice.

## 5 Biometric and scene-based analysis

Moving beyond the analysis of the digital file and its potential manipulations, forensic examiners often need to extract information from the visual content itself. This includes identifying individuals through biometric characteristics and measuring objects or events within the depicted scene. Recent years have seen a convergence of these disciplines, with deep learning providing a common technological foundation, while a strong push for scientific rigor and standardized reporting frameworks has reshaped how this evidence is presented in court.

### 5.1 Facial comparison and evidential value

Forensic facial comparison—the task of determining whether two images depict the same person—has undergone a significant methodological evolution. The practice is moving away from subjective, categorical conclusions (e.g., "it is a match") towards a more transparent and scientifically grounded probabilistic framework. The foundational methodology for comparison remains the Analysis, Comparison, Evaluation, and Verification (ACE-V) process, which provides a structured workflow for the examiner [51]. However, the critical development has been in the *Evaluation* stage, with the widespread adoption of the Likelihood Ratio (LR) framework for reporting the strength of evidence.

The LR framework, strongly recommended by bodies like ENFSI and ISO (ISO 21043). This framework provides a quantitative measure of the strength of the evidence that avoids the logical fallacies of absolute identification.

In practice, this is most often implemented through a score-based LR (SLR) systems. In this workflow, an automated facial recognition system, typically a deep learning model such as ArcFace or FaceNet, compares two images and outputs a numerical similarity score [52,53]. This score, on its own, is not forensically meaningful. It must be converted into an LR through a process called calibration. Calibration uses two distributions of scores—one from known "same-source" comparisons and one from known "different-source" comparisons—to model the probability of obtaining a given score under each hypothesis [52,54].

The validation of these SLR systems has become a major focus of research, with the Log-Likelihood-Ratio Cost (Cllr​) emerging as the standard performance metric [55]. Cllr​ is a powerful metric because it simultaneously evaluates both the discrimination (the ability of the system to separate same-source and different-source pairs) and the calibration (the accuracy of the probabilistic LR values) of the system [24,55]. A lower Cllr​ value indicates a better-performing, more reliable system. Recent research has focused on optimizing these systems. For instance, Macarulla Rodriguez et al. [24] showed that for surveillance videos, carefully selecting and pairing frames based on image quality and facial attributes (like pose) can significantly improve the resulting LRs and lower the Cllr​. Similarly Li et.al [45]. demonstrated that the LRs derived from modern deep learning features can be extremely powerful, providing strong evidence for identification.

The long-standing debate over human versus machine performance continues, but the landscape is shifting. While elite human forensic examiners and "super-recognizers" are highly accurate, studies show that the latest algorithms now perform at or even above the median level of human experts (NIST Face Recognition Technology Evaluation (FRTE), 2025), particularly on challenging, low-quality imagery [11]. The most accurate results are often achieved by merging the judgments of a human expert with the output of an algorithm, suggesting a future where AI acts as a powerful assistive tool rather than a replacement for the human examiner [11]. Though other studies pointed out that face comparison algorithms could also cause bias [56].

### 5.2 Forensic gait analysis

Forensic gait analysis, the identification of individuals based on their manner of walking, is another biometric modality that leverages video evidence. It is particularly valuable in cases where a suspect's face is obscured but their movement is captured on camera [57]. Analysis can be performed by examining a sequence of footprints or, more commonly, by observing video footage [58,59]. The process involves assessing various parameters, including spatial features like stride length and step width, and temporal features like cadence and joint angles [60,61].

Despite its use in some criminal trials, the scientific foundation of forensic gait analysis remains a subject of debate. There is an ongoing need for more extensive research to establish the uniqueness, consistency, and stability of gait features within and between individuals, which is necessary for its full acceptance as a reliable science [61,62].

Deep learning is increasingly being applied to automate gait recognition from video. Models are trained to extract gait features directly from video sequences or silhouettes, showing promise for improving accuracy [57,63,64]. However, the field faces significant challenges. A person's gait is not a fixed biometric; it is highly variable and can be affected by numerous intrinsic and extrinsic factors, including footwear, clothing, walking surface, speed, fatigue, injury, and even emotional state [58,62]. Furthermore, the quality of forensic analysis is heavily dependent on the quality of the video evidence, with factors like camera angle, resolution, and lighting posing major obstacles [58]. Recent validation studies have focused on assessing the accuracy of different measurement techniques, such as using wearable Inertial Measurement Units (IMUs) compared to gold-standard motion capture systems, with mixed results for different gait parameters [[65], [66], [67]].

### 5.3 Forensic photogrammetry

Forensic photogrammetry is the science of measurements from images and videos. It is a well-established discipline that applies principles of perspective geometry to reconstruct 3D information from 2D media, allowing examiners to determine the size, speed, and location of objects and people depicted in evidence [68]. Common photogrammetric methods include reverse projection photogrammetry (RPP), where a scene is revisited to take reference measurements, analytical photogrammetry, and 3D laser scanning [[68], [69], [70]].

A primary application of forensic photogrammetry is the estimation of vehicle speed from surveillance or dashboard camera footage [[71], [72], [73]]. The general workflow involves calibrating the camera to understand its geometric properties, detecting and tracking the vehicle across multiple frames, measuring the distance traveled in the 3D scene, and calculating the speed based on the time elapsed between frames (Xiao, & James, 2020; [73]).

Academic photogrammetry pipelines are heavily reliant on deep learning. Object detection models, most notably variants of YOLO (You Only Look Once), are used for robust vehicle detection, while tracking algorithms like DeepSORT or simple IOU-based trackers are used to follow the vehicle's trajectory through the video (Wang et al., 2024; [72]). Recent research in this area has focused significantly on improving computational efficiency to enable real-time analysis or deployment on resource-constrained edge devices. This includes the use of lightweight models (e.g., YOLOv6) and post-training quantization, which reduces the model's precision without a significant loss in accuracy [72].

As with any measurement science, reporting uncertainty is critical in forensic photogrammetry. An examiner's final report must include a margin of error for any calculated measurement, whether it be height, distance, or speed. Contributors to uncertainty are numerous and include the resolution of the imagery, lens distortion, the accuracy of reference measurements taken at the scene, and the stability of the video's frame rate [68,73]. The entire process is guided by comprehensive best practice documents from SWGDE and OSAC, which detail the requirements for evidence preparation, methodology, and the interpretation and reporting of results [68,69].

These three areas—facial comparison, gait analysis, and photogrammetry—demonstrate a clear convergence of disciplines within modern forensic video analysis. A single piece of video evidence may be subjected to all three types of analysis, often using a shared technological backbone. For instance, a deep learning object detector like YOLO can provide the initial bounding boxes needed for both vehicle speed estimation and human gait analysis. This integration creates a need for forensic analysts with broader, more integrated skill sets and underscores the importance of end-to-end validation of the entire forensic workflow, as errors in an early stage like object detection will inevitably propagate through all subsequent analyses. When using these methods strict validation is necessary.

## 6 Standardization and international collaboration

The rapid pace of technological change in forensic image and video analysis has created an urgent need for standardization. To ensure that evidence presented in court is reliable, repeatable, and scientifically valid, a global network of forensic science organizations, working groups, and law enforcement agencies has been actively developing and promoting best practices, guidelines, and formal standards. This collaborative effort is essential for bridging the gap between academic innovations and forensically sound applications, providing practitioners and laboratories with the frameworks needed to navigate the complexities of modern digital evidence.

A key trend in this area is the evolution from advisory "best practices" to more prescriptive, formal "standards." This shift is driven by increasing scrutiny of forensic evidence in legal systems worldwide and a broader movement across all forensic disciplines to strengthen their scientific foundations. Organizations like OSAC in the United States are working to place vetted standards on a public registry, making them the benchmark for laboratory accreditation and expert testimony [14,27]. This signifies a maturation of the field, where adherence to documented, consensus-based standards is becoming a mandatory requirement for quality assurance and legal admissibility. Table 3 summarizes the roles and recent contributions of the key international bodies shaping these standards.

**Table 3:** Key international working groups and standards bodies in forensic image/video analysis.

Organization/Body	Primary Focus Area	Key Recent Documents/Initiatives (2022-Present)	Relevance/Impact
**ENFSI** (European Network of Forensic Science Institutes)	Harmonization of forensic practices and quality management for member laboratories across Europe.	BPM for Digital Image Authentication (2022); Guideline for Facial Recognition System End Users (2023); BPM for Methodology of Speaker Comparison (2022); Research projects (e.g., S-FIVE).	Provides the framework for European laboratory accreditation under ISO/IEC 17025. BPMs are highly influential in establishing standard operating procedures.
**SWGDE** (Scientific Working Group on Digital Evidence)	Develop consensus-based best practices and guidelines for all aspects of digital and multimedia evidence.	Best Practices for Digital Video Authentication (2024); Best Practices for Image Content Analysis (2024); Position on Facial Recognition and Facial Comparison (2022); Best Practices for Photogrammetry (2022).	Documents are widely cited in North American courts and form the basis of many laboratory SOPs. Their open comment process ensures community input.
**FISWG** (Facial Identification Scientific Working Group)	Develop consensus standards, guidelines, and best practices specifically for image-based facial comparison.	Facial Identification Practitioner Code of Ethics (2024); Guideline for ACE-V Methodology in One-to-One Comparisons (2023); Principles for Responsible Use of Facial Recognition Technology (2024).	Defines the standard of practice for forensic facial comparison experts and provides critical guidance on methodology, ethics, and training.
**Interpol**	International law enforcement cooperation, managing global crime databases and promoting responsible technology use.	INTERPOL Facial Recognition System (IFRS); Policy Framework for Responsible Limits on Facial Recognition (with WEF & UNICRI) (2022); Guidelines for Digital Forensics First Responders.	Facilitates global criminal identification through its biometric databases. Promotes ethical and human-rights-compliant policies for law enforcement technology.
**EU Research Projects**	Funding and coordinating multi-national research to address specific forensic challenges and develop new technologies.	UNCOVER (Steganalysis); VISION (Forensic Biometrics); EXFILES (Mobile Forensics). ASGARD/STARLIGHT (AI use for LEAs) DETECT (detection of deep fakes) and many others	Drive innovation by combining academia, industry and law enforcement to create next-generation forensic tools and methodologies.

The **European Network of Forensic Science Institutes (ENFSI)** plays a pivotal role in harmonizing forensic practices across its European member laboratories. Through its Digital Imaging Working Group (DIWG) and Forensic IT working group (FITWG), ENFSI develops and publishes Best Practice Manuals (BPMs) that cover core areas of the discipline. Recent and relevant publications include the *BPM for Digital Image Authentication* [74], the *Guideline for Facial Recognition System End Users* [75], and earlier foundational documents on image enhancement and facial comparison [12,76]. These BPMs are crucial for laboratories seeking accreditation and serve as a benchmark for quality management.

The **Scientific Working Group on Digital Evidence (SWGDE)** is a leading voice in North America, producing an extensive library of guidelines and best practice documents through a consensus-based process that includes public comment periods [15]. Their publications cover nearly every topic in this review, with recent updates including *Best Practices for Digital Video Authentication* [77], *Best Practices for Image Content Analysis* [28], and a position paper on facial recognition [78]. These documents are highly influential and are frequently referenced in legal proceedings and used as the foundation for laboratory standard operating procedures.

Focusing specifically on the discipline of facial comparison, the **Facial Identification Scientific Working Group (FISWG)** brings together subject matter experts from law enforcement, intelligence agencies, and private industry to develop detailed standards [79]. Their recent work includes a *Facial Identification Practitioner Code of Ethics* [13], a *Guideline for ACE-V Methodology in One-to-One Comparisons* [51], and principles for the responsible use of facial recognition technology [13]. These documents provide the specific, granular guidance necessary for practitioners in this highly specialized field.

Globally, **Interpol** facilitates international law enforcement cooperation. Its INTERPOL Facial Recognition System (IFRS) is a unique global criminal database, populated with images from member countries and powered by technology from partners like IDEMIA [80,81]. Beyond operating this system, Interpol actively promotes the responsible use of such technologies. In collaboration with the World Economic Forum (WEF) and the United Nations Interregional Crime and Justice Research Institute (UNICRI), Interpol co-designed and published the "Policy Framework for Responsible Limits on Facial Recognition" in 2022, providing guidance to law enforcement on balancing security needs with human rights and privacy protections [16].

Finally, large-scale **European Union research projects**, often funded under programs like Horizon 2020, are critical drivers of innovation. Projects like **UNCOVER** are dedicated to developing advanced steganalysis tools for law enforcement [33,82]. These initiatives ensure that the European forensic community remains at the forefront of technological development while fostering collaboration between researchers and end-users.

## 7 Synthesis and future directions

The field of forensic image and video analysis is at a critical juncture, characterized by unprecedented technological capability matched by equally significant challenges of reliability and admissibility. The period from 2022 to the present has been defined by the pervasive influence of deep learning, which has simultaneously pushed the boundaries of what is possible while exposing the need for more robust validation and standardization. This review has charted the progress across key domains, revealing a discipline that is rapidly maturing.

Key trends have become clear. The dominance of deep learning is undeniable, reshaping everything from image enhancement and manipulation detection to biometric identification. This has triggered a necessary and critical re-evaluation of established techniques like PRNU, whose reliability is now questioned in the context of modern computational science. Concurrently, there has been a significant maturation in the evaluation of evidence, exemplified by the widespread adoption of the Likelihood Ratio framework in facial comparison, which moves the field towards a more transparent and scientifically defensible method of expressing conclusions. Underpinning all of these trends is the vital work of international bodies like ENFSI, SWGDE, and FISWG, whose efforts to create consensus-based standards are providing the essential guardrails for the responsible application of these powerful technologies.

Despite this progress, major challenges remain that will define the research and practice of the field for the foreseeable future. The most pressing of these is the issue of **Explainability and Interpretability (XAI)**. The "black box" nature of many deep learning models is fundamentally at odds with the legal system's requirement for transparency. For a forensic method to be admissible, an expert must be able to explain how it works and why its results are reliable. Future research must prioritize the development of models that are not only accurate but also transparent in their decision-making processes, allowing their internal logic to be understood [83,84].

A second major challenge is **Robustness and Generalization**. Many of the deep learning models reported in academic literature demonstrate impressive performance on clean benchmark datasets, but their effectiveness often plummets when applied to real-world "in-the-wild" evidence, which is typically of poor quality and has undergone multiple, unknown stages of compression and processing [30,37,38]. Bridging this gap between laboratory performance and operational reliability is important. This requires the development of models that are inherently more robust to degradation and the creation of more forensically realistic training and testing datasets [48,55].

This leads to the third challenge: **Data Scarcity and Privacy**. High-performance deep learning models are data-hungry, yet forensically relevant data is often highly sensitive, containing images of victims or suspects, and is subject to strict privacy and legal restrictions (for instance: the EU AI Act). This creates a bottleneck for research and development. There is a critical need for the creation of large-scale, diverse, and ethically sourced datasets that reflect the realities of casework. Furthermore, exploring privacy-preserving machine learning techniques, such as federated learning, where models can be trained across decentralized datasets without sharing the raw data itself, will be a crucial avenue for future research.

Finally, the **Pace of Counter-Forensics** ensures that the field will remain a dynamic arms race. As new methods for forgery or steganography detection are developed, adversaries will innovate new techniques to circumvent them. This requires the forensic science community to remain in a state of constant vigilance, with continuous research, development, and adaptation.

Looking ahead, the future of forensic image and video analysis will depend on addressing these challenges through concerted effort. The path forward requires the development of robust, explainable, and efficient AI models designed specifically for forensic use cases, not simply adapted from other computer vision domains. It demands continued research into novel biometric and device fingerprints that are resilient to the complexities of modern digital media pipelines. Most importantly, it necessitates even closer collaboration between academic researchers, forensic practitioners, standards bodies, and legal experts. By working together to bridge the validation gap, the community can ensure that the powerful innovations of today become the reliable, admissible, and justice-serving forensic tools of tomorrow.

## Declaration of competing interest

The authors declare that they have no known competing financial interests or personal relationships that could have appeared to influence the work reported in this paper.
